# Influence of Treatment Parameters on Beech Wood (*Fagus sylvatica*) Modified with Polyethylene Glycol and Various Carboxylic Acids

**DOI:** 10.3390/ma19091827

**Published:** 2026-04-29

**Authors:** Nicole Flaig, Melissa Christ, Marcus Müller

**Affiliations:** Material Development and Processing, University of Applied Forest Sciences Rottenburg, 72108 Rottenburg am Neckar, Germany; nicole_flaig@web.de (N.F.); christ@hs-rottenburg.de (M.C.)

**Keywords:** polyethylene glycol, carboxylic acids, optimisation, wood modification, treatment parameters

## Abstract

In this current study, beech wood (*Fagus sylvatica*) was modified by cross-linking via esterification with combinations of polyethylene glycol (PEG) 400 and various carboxylic acids. Promising combinations (1,2,3,4-butanetetracarboxylic acid (BTCA)/PEG400; citric acid (CA)/PEG400; malic acid (MA)/PEG400) were examined in previous studies. The goal of this study was the optimisation of the treatment. The use of a catalyst, the concentration of the chemicals and the curing conditions were varied. The weight percentage gain (WPG), bulking and anti-swelling efficiency (ASE) after leaching in water were used to evaluate the success of modification. Optimal results were achieved with a curing temperature of 160 °C. Without the addition of PEG, the WPG and bulking values were lower. The use of sodium hypophosphite monohydrate (SHP) as a catalyst had a positive effect only on the combination of BTCA/PEG400. Variations of concentrations usually had a higher impact on WPG and bulking than on ASE. The combination of MA/PEG 400 generally showed lower values.

## 1. Introduction

Research in the field of wood modification is mostly aimed at improving wood properties. Modified wood should be more dimensionally stable, less susceptible to wood-destroying fungi and more weather-resistant compared to untreated wood. The mechanical properties should not be changed or, in the best case, should be improved. Likewise, the modification process should be ecologically and economically feasible [1]. With this in mind, a modification with polyethylene glycol (PEG) and various acids, including citric acid (CA), was carried out in the current study. As already known from other studies, PEG and CA are water-soluble and no additional safety precautions are needed during the modification process [2,3]. Thus, this type of modification is characterised by a relatively low process effort. Another advantage is that lower emissions are produced both during the modification process and in the final product compared with, e.g., the widely researched chemical DMDHEU (1,3-dimethylol-4,5-dihydroxyethylene urea) [4].

The impregnation of wood with PEG was already investigated in the 1950s. PEGs are available in varying chain lengths and, as mentioned above, are highly water-soluble and have no dangerous goods labelling. Because of their outstanding properties, they also have various applications in everyday life, such as in pharmaceuticals and cosmetics [5].

In wood modification, PEG replaces water molecules in the cell walls, which causes a significant reduction in volumetric shrinkage after drying [6,7,8]. This bulking effect can lead not only to improved dimensional stabilisation but also to increased biological durability [9]. However, a main drawback of wood impregnation with PEG is that it is easily washed out with water due to its high water solubility and because it is not chemically anchored to the wood components [8].

Research has also been made regarding the esterification of wood components, such as cellulose, with polycarboxylic acids, mainly CA [1,10,11,12], partly also in combination with short polyols (such as sorbitol, glycerol and glucose) [13]. The addition of polyols should lead to a more flexible cross-linking network compared to one modified with acid alone [14]. Other than the addition of short polyols, a combination of CA/PEG600 has already been reported in one publication to modify paper cellulose [15]. These modifications have the advantage of chemical anchoring, and therefore have higher resistance against water leaching.

The cross-linking of cellulose with polycarboxylic acid has also been reported for butanetetracarboxylic acid (BTCA) [16,17,18], as well as the cross-linking of softwood kraft pulp fibres [19] and wood modification [20]. BTCA has an even higher cross-linking efficiency than CA, which can be explained by its tetrafunctionality (four carboxyl groups per molecule, which can possibly form ester bonds; for comparison, CA has three carboxyl groups) [20]. However, the high costs of BTCA may be a drawback for industrial application.

In many of the publications, the addition of sodium hypophosphite monohydrate (SHP) has been found to be efficient for the cross-linking of cellulose, e.g., with BTCA [10,18,20,21] and CA [1,4,18,20,21,22].

Few studies, however, can be found on the use of malic acid (MA) as a cross-linking agent for cellulose. This has primarily been investigated in relation to cotton fabrics [23,24]. The reason for that is probably that MA has only two carboxyl groups. The mechanism of cross-linking is believed to function via a five-membered anhydride, which would require at least three carboxyl groups for cross-linking (e.g., with cellulose) [16,25]. Based on this assumption, the existing cross-linking efficiency of cellulose with a dicarboxylic acid such as MA is surprising and was explained by the formation of a trimer of MA [24]. In a study by Chabert et al. [26], it was found that the leaching rate of beech wood treated with MA and glycerol improved when the MA was partially replaced by CA. The authors attributed this to the presence of a trifunctional molecule (CA) [26].

In a previous publication by the authors, screening experiments with combinations of PEG400 and different polycarboxylic acids for the modification of beech wood (*Fagus sylvatica*) were conducted [27]. PEG400 has a medium-sized chain length and has been found to have a good ability to diffuse into the cell wall [8]. The acids can form ester bonds both via cross-linking with the OH groups of PEG and wood components (cellulose). A highly improved resistance against water leaching was observed compared to impregnation with PEG alone, which can be explained by the chemical anchoring. Based on the positive results obtained there, it was decided to further investigate the combinations of BTCA/PEG400, CA/PEG400 and MA/PEG400 by the variation of different parameters, such as the presence of SHP as an additive, concentration and curing conditions. SHP has been used as a catalyst in wood modification with CA in many studies and tested at various concentrations [4,11,12,22,28,29]. Hasan et al. [4] found that higher SHP concentrations result in a reduction in ASE. He et al. [30] and Guo et al. [31] used only 1.5% and 1% SHP, respectively. In the current study, 3% SHP was used to avoid any potential negative effects caused by excessively high concentrations. Furthermore, it is expensive, not environmentally friendly and toxic substances such as hydrogen phosphide can be formed out of it [12,32].

## 2. Materials and Methods

### 2.1. Treatment

The concentration of chemicals for the impregnation solutions was calculated based on the weight of the chemical per volume of the entire solution. This means that in the specification “20% [*w*/*v*] of PEG400” denotes that in a water solution of 100 mL, there is 20 g of PEG400. The amount of acid in each impregnation solution was adjusted according to the amount of PEG400 so that the amount, i.e., the number of molecules, of acid was always twice the number of PEG400 molecules (molar ratio of acid:PEG400 = 2:1). An overview of the molar amounts of the different impregnation solutions is presented in Table 1. For each acid, a variant with half the chemical concentration was also tested. This generally requires fewer chemicals, which can result in a final product with a lower density and, consequently, a reduction in costs. In the designations of the different impregnation solutions, the number after the corresponding chemical (e.g., BTCA_10) denotes its concentration.

Different curing times of 3 h, 5 h, 7 h and 9 h (in the case of BTCA) and/or curing temperatures of 120 °C, 140 °C, 160 °C and 180 °C were selected for optimisation. Curing temperatures of 120 °C, 140 °C or 160 °C have often been used in the literature, particularly in relation to the modification of wood with CA [4,11,12,22,28]. A curing temperature of 180 °C has also been investigated [4,33]. Curing times of 10 h were predominantly tested [1,4,11,34], but also 8 h [29] or just 2.5 h [4]. Against this background, the corresponding curing temperatures were selected for this current study. With regard to duration, the times mentioned above were chosen in order to analyse the potential differing effects.

For all the experiments, PEG400 was obtained from S3 Chemicals (Bad Oeynhausen, Germany) and from Clariant (Munich, Germany)). SHP (“for analysis”) was purchased from Fisher Scientific (Schwerte, Germany). BTCA was obtained from Häberle (grade: “for synthesis”, Lonsee, Germany) and Fisher Scientific (98%, Schwerte, Germany). CA was purchased from Häberle (99.5%, water-free) and Carl Roth Chemicals (99.5%, water-free, Karlsruhe, Germany). MA was obtained from Fisher Scientific (98%) and Carl Roth Chemicals (≥99%).

### 2.2. Measurement of the pH Value

The pH value was determined for the combinations BTCA_10/PEG400_8.5/SHP_3, CA_19/PEG400_20 w/o cat. and MA_13.4/PEG400_20 w/o cat. In addition, untreated wood was added, which served as a control. For this purpose, 2.5 g of wood dust was generated in each case with the aid of a cutting mill with different mesh levels (4 mm, 1 mm, 0.25 mm). The pH values were measured using a mobile potentiometric pH meter. This was calibrated using the buffer solutions with pH values of 4 and 7. First, the pH of 50 mL of demineralised water was determined. Then, the respective wood dust was added and mixed for 15 min with a magnetic stirrer. Then, the pH meter was added to the solution and kept until a constant value was obtained. Two measurements were realised per variant.

### 2.3. Anti-Swelling Efficiency (ASE) Leaching Experiments

Specimens from beech sapwood with a size of 25 × 25 × 10 mm^3^ were used for the water leaching experiments. After the modification and curing of the specimens, ten cycles of weight-percent-gain (WPG), bulking and ASE were carried out for each of the ten specimens of one modification. For one cycle, the samples were placed in demineralised water and weighted with screw nuts to hinder that they float up, and a vacuum (about −0.9 bar) was applied for 30 min with an autoclave (Maschinenbau Scholz GmbH & Co. KG, Coesfeld, Germany).

The specimens were then kept at ambient pressure for another 24 h in water before measurement in wet conditions. Afterwards, the specimens were dried in the following mode: 24 h/room temperature (RT), 24 h/40 °C, 24 h/60 °C; 24 h/80 °C and 48 h/103 °C. The process was repeated a total of ten times for WPG, bulking and ASE. The tables listed in the results section each contain ASE values from cycle 1 to cycle 10, with the WPG and bulking values already starting at cycle 0.

The three key values, WPG, bulking and ASE, were calculated according to Equations (1)–(3):

(1) $$\mathrm{WPG}\;[\%]\;=\;\frac{m_{m}-m_{u}}{m_{u}}\cdot 100$$

m_m_ = Mass of the kiln-dried modified wood sample.

m_u_ = Mass of the kiln-dried unmodified wood sample.

(2) $$\mathrm{Bulking}\;[\%]=\frac{V_{m}-V_{u}}{V_{u}}\cdot 100$$

V_m_ = Volume of the kiln-dried modified wood sample.

V_u_ = Volume of the kiln-dried unmodified wood sample.

(3) $$\mathrm{ASE}\;[\%]=\frac{S_{u}-S_{m}}{S_{u}}\cdot 100\;\mathrm{with}\;S=\frac{V_{ws}-V_{kd}}{V_{kd}}\cdot 100$$

S_u_ = Swelling of the unmodified wood sample.

S_m_ = Swelling of the modified wood sample.

V_ws_ = Volume of the water-saturated wood sample.

V_kd_ = Volume of the kiln-dried wood sample.

## 3. Results and Discussion

As a basis for investigating the influence of the catalyst, the pH values of various combinations were examined. As can be seen in Table 2, the modified variants showed lower pH values compared to the control. However, the CA and MA variants were obviously more acidic than the BTCA combination. The control was slightly acidic and was in the range of 5.1 to 5.4, as reported in the literature [35].

In the next step, the influence of the effect of the additive, the concentration of the impregnation chemicals and the optimisation of the curing conditions were studied.

1. BTCA and combinations of BTCA/PEG400 in the molar ratio of 2:1.
2. CA and combinations of CA/PEG400 in the molar ratio of 2:1.
3. MA/PEG400 in the molar ratio of 2:1.

### 3.1. BTCA and BTCA/PEG400

In previous experiments, the beech wood specimens had been treated with an impregnation solution of BTCA (10% [*w*/*v*]) and PEG400 (8.5% [*w*/*v*]), without a catalyst (curing 5 h at 120 °C) [27]. The promising results from the screening experiments have prompted the authors to make further investigations to find the optimal conditions of the impregnation with solutions of BTCA:PEG400 in the molar ratio of 2:1.

#### 3.1.1. Effect of Catalyst

The influence of the addition of SHP as a catalyst on beech wood impregnation with solutions of BTCA (10% [*w*/*v*]):PEG400 (8.5% [*w*/*v*]) was investigated. The combination of BTCA, PEG and SHP almost always achieved the highest values, and the loss from cycles 0/1 to 10 was also lower (Table 3). In general, PEG400 clearly influenced the measured parameters, as the losses were highest without the addition of PEG. The catalyst influenced the values, especially WPG. This could be the result of increased cross-linking caused by the esterification reaction. The multifunctional nature of BTCA [36,37] likely promotes the formation of a three-dimensional cross-linked structure involving both cellulose hydroxyl groups and PEG chains, leading to increased dimensional stability [27]. However, the observed losses in WPG during leaching suggest that not all incorporated material is covalently bound, indicating a coexistence of chemically anchored and physically retained fractions.

The slightly positive effect on bulking was not observed for the experiments with combinations of PEG400 with CA and MA.

#### 3.1.2. Optimisation of the Curing Conditions

In contrast to CA and MA with decomposition temperatures of >161 °C and about 140 °C [38,39], BTCA was reported to be more temperature-stable up to 200 °C in the literature [40]. However, wood constituents (probably especially hemicelluloses) start to decompose earlier [41], which may also influence the results. Wang et al. [18] investigated the amount of esterified BTCA on cotton fabrics after curing at 120 °C, 140 °C, 160 °C and 180 °C with different curing times via titration. The authors found that an increase in temperature from 120 °C to 140 °C and further to 160/180 °C improved the consumption of BTCA. However, there was no major difference observed as to whether the curing was performed at 160 °C or 180 °C when comparing the results after equivalent reaction times, probably because of reaching an equilibrium condition [18].

For the BTCA/PEG systems, the focus was on a solution of BTCA (10% [*w*/*v*]) and PEG400 (8.5% [*w*/*v*]) with 3% [*w*/*v*] of SHP in demineralised water to find the optimal curing conditions. In the first step, again, curing conditions of 5 h at 140 °C, 160 °C and 180 °C were chosen and compared with the results after curing at 5 h/120 °C (Table 4). While improved results were observed after curing at 140 °C and 160 °C compared to curing of 5 h/120 °C, a curing of 5 h/180 °C delivered slightly lower values in bulking compared to curing at 160 °C. Although the absolute ASE values improved after curing for 5 h and at 180 °C instead of at 160 °C, their percentage loss after ten leaching cycles increased. Based on these results, the focus was on 160 °C as the curing temperature. The optimal temperature of 160 °C likely reflects a balance between increased reaction kinetics and the onset of the thermal degradation of wood constituents. At lower temperatures, insufficient activation limits esterification, whereas at higher temperatures, the degradation of hemicelluloses and potential structural damage reduce the effectiveness of the modification [13,42,43].

Then, further experiments were conducted to explore the influence of the curing time; curing conditions of 3 h, 5 h, 7 h and 9 h/160 °C were compared. The influence of the curing time turned out to be minimal.

#### 3.1.3. Influence of the Concentration of the Impregnation Solutions

The results after leaching for beech wood impregnated with a solution of 10% [*w*/*v*] of BTCA and 8.5% [*w*/*v*] of PEG400 with 3% [*w*/*v*] of the catalyst SHP were compared with the results of beech wood that had been impregnated with two solutions containing a reduced amount of the chemicals. In the first, BTCA, PEG and SHP were reduced, whereas in the second, PEG was omitted altogether (Table 5). The values of the wood impregnated with 5% [*w*/*v*] of BTCA, 4.3% [*w*/*v*] of PEG400 and 1.5% [*w*/*v*] of SHP reached only about half the value of the wood impregnated with a higher solution concentration at the beginning of the measurement of bulking and WPG. Reducing the concentration led to higher losses, likely due to insufficient chemical content for effective network formation.

The addition of PEG clearly influenced the measured parameters. This is because the values were also obviously lower at the beginning without the addition of PEG. Furthermore, the losses were highest from cycles 0 to 10 without the use of PEG. This was also the case for CA/PEG400 (Table 6). PEG is one of the chemicals that can lead to considerable bulking [9]. The results suggest that PEG is primarily responsible for the high bulking values.

The ASE values of the beech wood specimens impregnated with the lower concentrated solutions and PEG were lower, too, but not as remarkable as in the case of WPG and bulking. Additionally, the percentage losses in ASE of the higher and lower concentrated variants were comparable from cycle 1 to 10. The specimens with the lower concentrated solution can certainly keep up with the samples with the higher concentrated solution. As already mentioned for WPG and bulking, the loss from cycle 1 to 10 was highest for the ASE of the lower concentrated variant without PEG. Up to cycle 5, however, the values were relatively comparable with those of the lower concentrated variant with PEG. Thus, the influence of PEG on ASE was also less pronounced here than for WPG and bulking.

### 3.2. CA and CA/PEG400

In preliminary screening experiments, the authors found promising results with impregnation solutions that consisted of CA (38% [*w*/*v*]) and PEG400 (40% [*w*/*v*]), without a catalyst (curing 5 h at 120 °C) [27]. In the following experiments, starting from this solution concentration (molar ratio of CA:PEG400 of 2:1), an attempt was made to find the optimal conditions for impregnation.

#### 3.2.1. Effect of Catalyst

The addition of 3% [*w*/*v*] of SHP to an impregnation solution of 38% [*w*/*v*] of CA and 40% [w/v] of PEG400 had a negative effect on ASE and bulking, but not on WPG after leaching (Table 6). In contrast, the influence of SHP addition to a solution of 38% [*w*/*v*] of CA without PEG was positive on bulking and WPG. The pronounced negative effect of SHP on the impregnation with 38% [*w*/*v*] of CA and 40% [*w*/*v*] PEG400 was in contrast to the positive influence of SHP on the corresponding BTCA and BTCA/PEG400 solutions (Table 3).

In the literature, different effects of SHP on the results of cellulose esterification have been reported. Hasan et al. [4] investigated the effects of different concentrations of SHP on the modification of beech wood with CA, e.g., on ASE, and the lowest SHP concentration showed the best ASE results. Feng et al. [12] investigated the effect of the addition of 5% of SHP (based on CA mass) on the esterification of poplar wood with CA. The authors observed only very slightly higher WPG, bulking and ASE results for the catalysed system [12]. Cuadro et al. [15] impregnated filter paper with water solutions of 20 wt% of CA and 10 wt% of PEG600. They even observed a 3.5 fold increase in WPG with an additional 3 wt% of SHP than without. In contrast, they also reported that with a water solution of only 5 wt% of CA and 10 wt% of PEG600, the effect of the addition of 3 wt% on WPG of the filter paper was not significant [15].

The addition of 3% [*w*/*v*] of SHP to the system of CA (38% [*w*/*v*]):PEG400 (40% [*w*/*v*]) led to a remarkable decrease in ASE in leaching experiments. The underlying mechanism for this behavior remains unclear. Feng et al. [12] emphasise that the mechanism of action and the results of SHP have not yet been fully clarified. Furthermore, the role of SHP is controversial. It has been observed that SHP may react competitively with anhydride, and that the resulting stable acylphosphinates reduce the degree of esterification [12]. Morris et al. [44] point out that the catalytic effect of hypophosphite on the reaction between anhydride and cellulose remains unclear.

Perhaps the added SHP changed the ratio of esterified CA/PEG400 and therefore the ASE properties of the resulting impregnated wood. It is also possible that the low pH value is not in the optimal range for the esterification mechanism, which is believed to occur via the formation of an anhydride of the acid and subsequent reaction with cellulose [13,45]. While lower pH values favour anhydride formation, excessively acidic conditions may hinder subsequent reactions with cellulose or promote hydrolytic degradation, thereby reducing overall modification efficiency. CA can often create highly acidic conditions, causing the reaction to become atuocatalytic [12]. Ji et al. [45,46] investigated the effect of the pH on the esterification of cellulose with BTCA in the presence of different alkaline salts as catalysts. They found out that on the one side, a lower pH accelerates the anhydride formation, but on the other side, a higher pH (higher anion concentration of the catalyst) accelerates the reaction with cellulose in the final step of esterification reaction [45,46]. In the case of BTCA with SHP as the catalyst, promising results have been obtained by Welch and Andrews [47] and Yang [48]. Therefore, it could be that the pH value of the combination CA, PEG and SHP was not suitable. One has to keep in mind that the pH of a BTCA solution always increases when SHP is added since SHP has a slightly alkaline character [49]. In the current study, only the BTCA combination contained SHP in the pH measurement. It is possible that the higher value is also due to its addition.

#### 3.2.2. Influence of the Concentration of the Impregnation Solution

Moreover, the influence of the concentration of the impregnation solution on the key parameters was investigated. Therefore, the results after impregnation with a solution of 38% [*w*/*v*] of CA and 40% [*w*/*v*] of PEG400 (molar ratio CA/PEG400 = 2:1) in demineralised water (values taken from previous experiments [27]) were compared to the results after impregnation with a lower solution concentration, namely 19% [*w*/*v*] CA and 20% [*w*/*v*] PEG400 (Table 6). It could be seen that the bulking and WPG values of the impregnation with higher solution concentration were noticeably higher. As is already described in detail in the literature [4,13,15], it seems plausible that as the concentration of chemicals increases, the weight-percentage-gain also increases.

Interestingly, the ASE values of the combination 19% [*w*/*v*] CA and 20% [*w*/*v*] PEG400 were even higher in some cases. Similar observations were already made in preliminary screening experiments [27]. However, the reason for this remains unclear. In turn, this combination is characterised by higher losses in ASE from cycle 1 to 10. Nevertheless, in both cases, the modification was successful since the ASE after ten cycles is still high, at about 39% for the combination with 38% [*w*/*v*] of CA and 40% [*w*/*v*] of PEG400, and at about 31% for the lower solution concentration. Although the loss of bulking after ten cycles is higher for the combination with the lower concentrated solution, the values are still suitable after the end of the measurement. This and also the high WPG values indicate that the chemicals are fixed in the wood. It should also be noted that without the addition of PEG, higher losses occurred from cycles 0/1 to 10 for all three key values. Perhaps in this case the acids are less fixed in the wood.

At present, there is no evidence to suggest that cross-linking between CA and the wood components actually occurs [50]. However, Christ et al. [51] found indices of cross-linking between acid, PEG and wood components by investigating the impact bending strength and elongation. The impact bending strength has decreased significantly, which could be attributed to the cross-linked structure that increases the stiffness of the wood and hinders the free movement of the microfibrils [52]. Elongation also decreased, suggesting a cross-linked wood structure.

**Table 6:** Influence of the concentration of chemicals and the addition of 3% [*w*/*v*] of SHP on ASE, bulking and WPG values in leaching experiments with beech wood ASE samples impregnated with water solutions of CA/PEG400 in molar ratios of 2:1 (curing conditions: 5 h/120 °C). Standard deviations are given in brackets (w/o = without; cat. = catalyst).

Parameter and Formulation	0	Cycle 1	Cycle 5	Cycle 10	Loss [%] 0/Cycle 1 to Cycle 10
ASE [%] (CA_38/PEG400_40) w/o cat.		54.73 (5.18)	37.34 (5.39)	39.46 (2.70)	−27.9
ASE [%] (CA_38/PEG400_40/SHP_3)		30.69 (5.18)	26.10 (5.27)	20.48 (5.27)	−33.3
ASE [%] (CA_38) w/o PEG w/o cat.		54.86 (3.12)	36.94 (4.29)	32.53 (1.94)	−40.7
ASE [%] (CA_38/SHP_3) w/o PEG		59.09 (1.78)	43.58 (2.77)	35.63 (2.52)	−39.7
ASE [%] (CA_19/PEG400_20) w/o cat.		65.54 (3.23)	41.29 (3.79)	31.23 (3.84)	−52.3
Bulking [%] (CA_38/PEG400_40) w/o cat.	20.58 (1.20)	18.60 (1.32)	17.21 (1.48)	16.80 (1.53)	−18.4
Bulking [%] (CA_38/PEG400_40/SHP_3)	14.10 (2.04)	12.78 (1.98)	12.32 (2.11)	11.39 (2.19)	−19.2
Bulking [%] (CA_38) w/o PEG w/o cat.	12.76 (0.40)	8.70 (0.51)	6.40 (0.54)	5.25 (0.62)	−58.9
Bulking [%] (CA_38/SHP_3) w/o PEG	12.78 (0.56)	10.73 (0.57)	9.07 (0.55)	7.25 (0.48)	−43.3
Bulking [%] (CA_19/PEG400_20) w/o cat.	15.52 (0.40)	11.03 (0.66)	10.44 (0.69)	9.69 (0.78)	−37.6
WPG [%] (CA_38/PEG400_40) w/o cat.	59.74 (7.50)	42.91 (1.53)	38.63 (1.39)	36.95 (1.31)	−38.1
WPG [%] (CA_38/PEG400_40/SHP_3)	57.12 (5.35)	48.48 (4.66)	43.02 (4.42)	40.60 (4.34)	−28.9
WPG [%] (CA_38) w/o PEG w/o cat.	35.26 (2.26)	25.47 (1.22)	17.91 (0.94)	13.14 (1.01)	−62.7
WPG [%] (CA_38/SHP_3) w/o PEG	31.22 (1.14)	24.91 (0.75)	18.88 (0.70)	14.75 (0.59)	−52.8
WPG [%] (CA_19/PEG400_20) w/o cat.	35.68 (3.14)	27.56 (2.50)	23.61 (2.23)	21.97 (2.19)	−38.4

#### 3.2.3. Optimisation of the Curing Conditions

For the impregnation system of CA (19% [*w*/*v*]):PEG400 (20% [*w*/*v*]) (molar ratio of 2:1) without a catalyst, the leaching results after curing for 5 h at 120 °C, 140 °C, 160 °C and 180 °C were compared.

Curing conditions on wood that has been modified by CA have already been compared in the literature. Beech wood that had been treated with mixtures of CA and glycerol was cured for 72 h at 103 °C, 120 °C, 140 °C and 160 °C. The authors stated that both the leaching stability and the ASE increased with increasing temperatures, especially when higher than 140 °C. However, WPG decreased significantly after curing at 160 °C compared to curing at 140 °C [28]. Leaching experiments on pine wood that had been esterified with CA and glycerol have shown that curing at 18 h/103 °C was not enough to be leaching-resistant when compared to curing at 140 °C, which led to higher WPG stability after leaching [53].

The tendency of the current curing experiments with CA/PEG400 concerning bulking and WPG turned out to be comparable to the results with BTCA/PEG and MA/PEG. The values improved up to 160 °C, but decreased after curing at 180 °C. The ASE values after curing at 180 °C were slightly lower than after curing at 160 °C, too, but the percentage loss was improved (Table 7). One reason for this could be the thermolysis of wood components [41]. With regard to the stability of the modification, the largest losses of cycle 0/1 to 10 were observed for ASE, WPG and bulking for the curing conditions of 5 h/120 °C.

### 3.3. MA/PEG400

In previous screening experiments, it was found that the combination of 27% [*w*/*v*] of MA and 40% [*w*/*v*] of PEG400 (molar ratio MA:PEG400 = 2:1) without a catalyst (curing conditions 5 h/120 °C) was promising [27]. The focus was now set on the optimisation of parameters such as the influence of SHP as a catalyst, the concentration of the chemicals in a solution and the curing conditions.

#### 3.3.1. Effect of Catalyst

The addition of 3% [*w*/*v*] of SHP showed a negative effect on ASE and bulking values compared to the uncatalysed system for beech wood ASE specimens impregnated with a solution of MA (27% [*w*/*v*]):PEG400 (40% [*w*/*v*]) (Table 8). However, this negative influence of the SHP, especially on ASE, was not so pronounced as for the CA/PEG400 system (Table 6). As written in Section 3.2.1, different experimental results and theories have already been published on the catalytic effect of SHP [12,45,46], and the outcome might depend on whether the pH value was in the optimal range. Thus, a pH measurement revealed that the CA and MA variants were in a similar low pH range (Table 2). This could explain the different effects observed after SHP addition on the systems BTCA/PEG400, CA/PEG400 and MA/PEG400.

In general, the lower performance of MA can be attributed to the limited number of carboxyl groups, which restricts the formation of anhydride intermediates and reduces the probability of effective cross-linking [13,54,55]. Conversely, the intermediate behaviour of CA reflects its trifunctional nature [56], which enables network formation.

#### 3.3.2. Effect of the Concentration of Chemicals in Solution

As for the systems of BTCA/PEG400 and CA/PEG400, first, the effect of a lower solution concentration, starting from the solution of 27% [*w*/*v*] of MA and 40% [*w*/*v*] of PEG400 [27], was investigated.

Compared with the combination with a higher solution concentration (27% [*w*/*v*] of MA and 40% [*w*/*v*] of PEG400) without a catalyst, the losses of the reduced solution concentration (13.5% [*w*/*v*] of MA and 20% [*w*/*v*] of PEG400) were higher after ten cycles for all three key parameters, with the highest loss seen in bulking (Table 8). In general, the values over the cycles were always lower than for the higher solution concentration. The possible reason for this has already been explained in Section 3.1.3.

#### 3.3.3. Optimisation of the Curing Conditions

Experiments to optimise the curing conditions were also conducted for MA/PEG400. Therefore, the focus was on one specific recipe of the impregnation solution, namely a solution of MA (13.4% [*w*/*v*]) and PEG400 (20% [*w*/*v*]) without a catalyst. As for the other systems, curing temperatures of 140 °C, 160 °C and 180 °C with curing times of 5 h were tested.

The results of the leaching experiments are shown in Table 9 in comparison to the results with curing conditions of 5 h/120 °C. For WPG and bulking, an increase in curing temperature resulted on average in better values up to 160 °C, while the ASE results stayed relatively constant between 140 °C and 180 °C after an improvement compared to 120 °C. For WPG, predominantly improved values were obtained at a curing of 160 °C and 180 °C compared to the curing conditions of 120 °C for 5 h. The losses from cycles 0 to 10 were also lower. However, the WPG and bulking values decreased at 180 °C compared to the ones after curing at 160 °C. This phenomenon has already been observed for the system BTCA/PEG400 and CA/PEG400. As for the MA system, the largest losses always occurred from cycles 0/1 to 10 for curing conditions of 5 h/120 °C for ASE, WPG and bulking. Overall, the losses from cycles 0 to 10 of the bulking were most affected by the different temperatures, as was the case for BTCA/PEG400 and CA/PEG400. Nevertheless, it was noticeable that the losses, regardless of the curing temperature, were always higher than those of the BTCA and CA combinations. In a study by Chabert et al. [26], the dimensional stability of beech wood treated with MA and glycerol was investigated. Both ASE and bulking values have been improved. The leaching tests showed that the combination of MA and glycerol had a higher leaching rate compared to the variant in which the MA was partially replaced by CA. The authors assume that the presence of a trifunctional molecule (CA) improves the efficacy of the treatment [26].

Finally, the correlation between WPG, bulking and ASE across different systems has demonstrated that a high initial mass gain does not necessarily translate into improved stability, and that effective cross-linking—rather than total uptake—is the key factor governing dimensional stability.

## 4. Conclusions

In this current study, wood modifications with combinations of BTCA, CA and MA with PEG400 in the molar ratio of 2:1 were optimised. The following parameters were varied: the addition of a catalyst, the concentration of the chemicals in solution and the curing conditions. The modifications were evaluated by leaching tests in water.

In the range of 120 °C to 180 °C, curing conditions of 160 °C in general turned out to give the most promising results. Optimum polymerisation/cross-linking of the components has probably taken place. Especially, the losses of the bulking after ten cycles were improved by the optimised conditions (5 h/160 °C). The incorporation of PEG400 resulted in higher WPG and bulking values. Without the addition of PEG, these values were lower. It seems that the PEG is predominantly responsible for the high bulking values since it can lead to considerable bulking anyway. In addition, the losses due to leaching were much higher in WPG and bulking without the presence of PEG. This indicates that PEG plays a critical role in bulking and WPG. The addition of 3% [*w*/*v*] of SHP to the impregnation solution showed a positive effect only with the use of BTCA, whereas no effect was detected for CA/PEG400 and MA/PEG400. It is possible that the measured, comparatively lower pH values of the CA and MA combinations were not in the optimal range for the esterification mechanism, which is believed to happen via the formation of an anhydride of the acid and subsequent reaction with cellulose. It could be shown that the percentage decrease of WPG and bulking was often comparable to the decrease in concentration, while the effect on ASE was lower. It seems plausible that as the concentration of chemicals increases, the WPG also increases, and vice versa. Perhaps the lower chemical concentration resulted in less cross-linking. The underlying reason for this behavior remains unclear.

## Figures and Tables

**Table 1:** List of molar amounts of the impregnation solutions (cat. = catalyst; w/o = without).

Formulation	n (Acid) [mmol]	n (PEG400) [mmol]	n (Cat.) [mmol]
BTCA_10 w/o PEG w/o cat.	213.5		
BTCA_10/SHP_3 w/o PEG	213.5		144.3
BTCA_10/PEG400_8.5 w/o cat.	213.5	106.8	
BTCA_10/PEG400_8.5/SHP_3	213.5	106.8	144.3
BTCA_5/SHP_1.5 w/o PEG	64.1		43.3
BTCA_5/PEG400_4.3/SHP_3	64.1	32.0	86.6
BTCA_5/PEG400_4.3/SHP_1.5	64.1	32.0	43.3
CA_38 w/o PEG w/o cat.	1665.6		
CA_38/SHP_3 w/o PEG	1041.0		144.3
CA_38/PEG400_40 w/o cat.	600.0	300.0	
CA_38/PEG400_40/SHP_3	1000.0	500.0	144.3
CA_19/PEG400_20 w/o cat.	1200.0	600.0	
MA_27/PEG400_40 w/o cat.	600.0	300.0	
MA_27/PEG400_40/SHP_3	1000.0	500.0	144.3
MA_13.4/PEG400_20 w/o cat.	350.0	175.0	

**Table 2:** pH values of selected modifications (demin. = demineralised; w/o = without; cat. = catalyst).

Formulation	pH (Demin. Water and Wood)
Control	5.1 5.1
BTCA_10/PEG400_8.5/SHP_3	4.8 4.8
CA_19/PEG400_20 w/o cat.	3.4 3.4
MA_13.4/PEG400_20 w/o cat.	3.1 3.1

**Table 3:** Influence of the addition of 3% [*w*/*v*] of SHP on ASE, bulking and WPG values in leaching experiments with beech wood ASE samples impregnated with water solutions of either BTCA (10% [*w*/*v*]):PEG400 (8.5% [*w*/*v*]) (molar ratio of 2:1) or BTCA (10% [*w*/*v*]) without PEG (curing conditions: 5 h/120 °C). Standard deviations are given in brackets (w/o = without; cat. = catalyst).

Parameter and Formulation	0	Cycle 1	Cycle 5	Cycle 10	Loss [%] 0/Cycle 1 to Cycle 10
ASE [%] (BTCA_10/PEG400_8.5) w/o cat.		48.85 (5.34)	41.12 (2.98)	37.43 (3.69)	−23.4
ASE [%] (BTCA_10/PEG400_8.5/SHP_3)		49.02 (2.54)	41.97 (2.97)	36.91 (3.34)	−24.7
ASE [%] (BTCA_10) w/o PEG w/o cat.		38.54 (5.32)	27.78 (5.00)	25.32 (5.06)	−34.3
ASE [%] (BTCA_10/SHP_3) w/o PEG		49.27 (4.90)	42.28 (3.30)	37.26 (2.74)	−32.2
Bulking [%] (BTCA_10/PEG400_8.5) w/o cat.	6.89 (0.82)	4.28 (0.74)	5.47 (0.50)	5.36 (0.56)	−22.2
Bulking [%] (BTCA_10/PEG400_8.5/SHP_3)	8.11 (0.79)	7.26 (0.95)	7.31 (0.90)	6.37 (0.93)	−21.5
Bulking [%] (BTCA_10) w/o PEG w/o cat.	4.15 (0.56)	1.87 (0.38)	2.80 (0.61)	2.32 (0.61)	−44.1
Bulking [%] (BTCA_10/SHP_3) w/o PEG	4.87 (0.89)	3.44 (0.50)	3.42 (0.64)	2.97 (0.82)	−39.0
WPG [%] (BTCA_10/PEG400_8.5) w/o cat.	17.15 (1.22)	13.76 (1.15)	12.01 (1.08)	10.76 (1.02)	−37.7
WPG [%] (BTCA_10/PEG400_8.5/SHP_3)	18.33 (1.10)	15.33 (0.88)	13.84 (0.90)	12.68 (0.74)	−30.8
WPG [%] (BTCA_10) w/o PEG w/o cat.	8.98 (0.83)	5.46 (0.61)	3.97 (0.59)	2.97 (0.64)	−66.9
WPG [%] (BTCA_10/SHP_3) w/o PEG	11.74 (0.92)	8.19 (0.80)	6.52 (0.68)	5.28 (0.73)	−55.0

**Table 4:** Influence of the curing conditions on ASE, bulking and WPG values in leaching experiments with beech wood ASE samples impregnated with water solutions of BTCA (10% [*w*/*v*]):PEG400 (8.5% [*w*/*v*]) (molar ratio of 2:1) with 3% [*w*/*v*] SHP. Standard deviations are given in brackets.

Parameter and Formulation	0	Cycle 1	Cycle 5	Cycle 10	Loss [%] 0/Cycle 1 to Cycle 10
ASE [%] (BTCA_10/PEG400_8.5/SHP_3) 5 h/120 °C		49.02 (2.54)	41.97 (2.97)	36.91 (3.34)	−24.7
ASE [%] (BTCA_10/PEG400_8.5/SHP_3) 5 h/140 °C		50.35 (2.82)	47.32 (2.68)	41.12 (2.94)	−22.4
ASE [%] (BTCA_10/PEG400_8.5/SHP_3) 3 h/160 °C		52.41 (1.98)	46.36 (3.17)	42.10 (2.05)	−19.7
ASE [%] (BTCA_10/PEG400_8.5/SHP_3) 5 h/160 °C		53.66 (2.15)	46.10 (2.68)	40.90 (1.98)	−23.8
ASE [%] (BTCA_10/PEG400_8.5/SHP_3) 7 h/160 °C		54.17 (2.66)	47.77 (3.42)	43.20 (2.62)	−25.4
ASE [%] (BTCA_10/PEG400_8.5/SHP_3) 9 h/160 °C		52.74 (2.36)	48.35 (3.11)	43.81 (5.05)	−16.9
ASE [%] (BTCA_10/PEG400_8.5/SHP_3) 5 h/180 °C		60.06 (2.39)	52.01 (3.92)	45.97 (3.11)	−30.7
Bulking [%] (BTCA_10/PEG400_8.5/SHP_3) 5 h/120 °C	8.11 (0.79)	7.26 (0.95)	7.31 (0.90)	6.37 (0.93)	−21.5
Bulking [%] (BTCA_10/PEG400_8.5/SHP_3) 5 h/140 °C	8.62 (0.98)	8.36 (0.92)	7.84 (1.04)	7.87 (1.08)	−8.7
Bulking [%] (BTCA_10/PEG400_8.5/SHP_3) 3 h/160 °C	9.13 (0.44)	8.29 (0.37)	8.13 (0.62)	7.72 (0.60)	−15.4
Bulking [%] (BTCA_10/PEG400_8.5/SHP_3) 5 h/160 °C	8.64 (0.55)	8.67 (0.70)	8.24 (0.47)	8.04 (1.02)	−6.9
Bulking [%] (BTCA_10/PEG400_8.5/SHP_3) 7 h/160 °C	9.43 (0.70)	8.55 (0.91)	8.45 (0.48)	7.95 (---)	−15.7
Bulking [%] (BTCA_10/PEG400_8.5/SHP_3) 9 h/160 °C	9.03 (0.59)	8.51 (0.63)	8.41 (0.46)	7.07 (0.41)	−21.7
Bulking [%] (BTCA_10/PEG400_8.5/SHP_3) 5 h/180 °C	7.17 (0.80)	7.31 (0.82)	6.21 (0.56)	6.16 (0.84)	−14.1
WPG [%] (BTCA_10/PEG400_8.5/SHP_3) 5 h/120 °C	18.33 (1.10)	15.33 (0.88)	13.84 (0.90)	12.68 (0.74)	−30.8
WPG [%] (BTCA_10/PEG400_8.5/SHP_3) 5 h/140 °C	17.85 (0.40)	15.57 (0.49)	14.04 (0.28)	13.01 (0.40)	−27.1
WPG [%] (BTCA_10/PEG400_8.5/SHP_3) 3 h/160 °C	17.30 (0.53)	16.78 (0.80)	15.01 (0.58)	13.63 (1.05)	−21.2
WPG [%] (BTCA_10/PEG400_8.5/SHP_3) 5 h/160 °C	16.31 (0.51)	15.68 (0.60)	13.87 (0.61)	12.81 (0.60)	−21.5
WPG [%] (BTCA_10/PEG400_8.5/SHP_3) 7 h/160 °C	17.18 (0.59)	16.70 (0.53)	15.23 (0.56)	13.64 (1.82)	−20.6
WPG [%] (BTCA_10/PEG400_8.5/SHP_3) 9 h/160 °C	16.79 (0.69)	16.39 (0.63)	14.97 (0.48)	13.35 (0.74)	−20.5
WPG [%] (BTCA_10/PEG400_8.5/SHP_3) 5 h/180 °C	15.66 (1.44)	15.23 (1.48)	13.38 (1.40)	12.00 (1.34)	−23.4

**Table 5:** Influence of the concentration of chemicals on WPG, bulking and ASE values in leaching experiments with beech wood ASE samples impregnated with water solutions of BTCA and BTCA/PEG400 (molar ratio 2:1) and 1.5 or 3% [*w*/*v*] of SHP (curing conditions: 5 h/160 °C). Standard deviations are given in brackets (w/o = without).

Parameter and Formulation	0	Cycle 1	Cycle 5	Cycle 10	Loss [%] 0/Cycle 1 to Cycle 10
ASE [%] (BTCA_10/PEG400_8.5/SHP_3)		53.66 (2.15)	46.10 (2.68)	40.90 (3.69)	−23.8
ASE [%] (BTCA_5/PEG400_4.3/SHP_1.5)		41.19 (2.19)	32.68 (2.96)	29.74 (3.82)	−27.8
ASE [%] (BTCA_5/SHP_1.5) w/o PEG		41.70 (1.15)	31.11 (2.62)	23.23 (1.68)	−44.3
Bulking [%] (BTCA_10/PEG400_8.5/SHP_3)	8.64 (0.55)	8.67 (0.70)	8.24 (0.47)	8.04 (1.20)	−7.5
Bulking [%] (BTCA_5/PEG400_4.3/SHP_1.5)	4.61 (0.37)	3.71 (0.30)	3.21 (0.36)	3.15 (0.38)	−31.7
Bulking [%] (BTCA_5/SHP_1.5) w/o PEG	1.85 (0.17)	1.46 (0.27)	1.37 (0.36)	0.93 (0.41)	−49.7
WPG [%] (BTCA_10/PEG400_8.5/SHP_3)	16.31 (0.51)	15.68 (0.60)	13.87 (0.61)	12.81 (0.60)	−21.5
WPG [%] (BTCA_5/PEG400_4.3/SHP_1.5)	7.69 (0.55)	7.54 (0.54)	5.67 (0.77)	5.32 (0.48)	−30.8
WPG [%] (BTCA_5/SHP_1.5) w/o PEG	4.53 (0.44)	4.16 (0.29)	2.80 (0.20)	1.32 (0.25)	−70.9

**Table 7:** Influence of the curing conditions on ASE, bulking and WPG values in leaching experiments with beech wood ASE samples impregnated with water solutions of CA (19% [*w*/*v*]):PEG400 (20% [*w*/*v*]) (molar ratio of 2:1) without a catalyst. Standard deviations are given in brackets (w/o = without; cat. = catalyst).

Parameter and Formulation	0	Cycle 1	Cycle 5	Cycle 10	Loss [%] 0/Cycle 1 to Cycle 10
ASE [%] (CA_19/PEG400_20) w/o cat. 5 h/120 °C		65.54 (3.23)	41.29 (3.79)	31.23 (3.84)	−52.3
ASE [%] (CA_19/PEG400_20) w/o cat. 5 h/140 °C		56.73 (3.26)	39.77 (3.07)	37.65 (3.74)	−33.6
ASE [%] (CA_19/PEG400_20) w/o cat. 5 h/160 °C		63.29 (2.28)	50.43 (3.04)	43.12 (4.38)	−31.9
ASE [%] (CA_19/PEG400_20) w/o cat. 5 h/180 °C		58.68 (1.43)	46.63 (2.17)	42.19 (1.28)	−28.1
Bulking [%] (CA_19/PEG400_20) w/o cat. 5 h/120 °C	15.52 (0.40)	11.03 (0.66)	10.44 (0.69)	9.69 (0.78)	−37.6
Bulking [%] (CA_19/PEG400_20) w/o cat. 5 h/140 °C	15.98 (0.65)	14.10 (0.61)	12.75 (0.59)	12.63 (0.64)	−21.0
Bulking [%] (CA_19/PEG400_20) w/o cat. 5 h/160 °C	13.97 (0.98)	13.56 (1.01)	12.23 (0.90)	11.72 (0.98)	−16.1
Bulking [%] (CA_19/PEG400_20) w/o cat. 5 h/180 °C	10.53 (1.11)	9.91 (1.14)	8.33 (0.92)	7.98 (1.08)	−24.2
WPG [%] (CA_19/PEG400_20) w/o cat. 5 h/120 °C	35.68 (3.14)	27.56 (2.50)	23.61 (2.23)	21.97 (2.19)	−38.4
WPG [%] (CA_19/PEG400_20) w/o cat. 5 h/140 °C	31.46 (1.02)	28.52 (0.95)	25.58 (0.86)	23.74 (1.03)	−24.5
WPG [%] (CA_19/PEG400_20) w/o cat. 5 h/160 °C	33.16 (3.55)	32.08 (3.49)	29.72 (3.46)	28.10 (3.35)	−15.3
WPG [%] (CA_19/PEG400_20) w/o cat. 5 h/180 °C	26.13 (2.95)	25.08 (3.04)	22.90 (2.77)	21.41 (2.79)	−18.1

**Table 8:** Influence of the concentration of chemicals and of the addition of 3% [*w*/*v*] of SHP on ASE, bulking and WPG values in leaching experiments with beech wood ASE samples impregnated with water solutions of MA and PEG400 in a molar ratio of 2:1 (curing conditions: 5 h/120 °C). Standard deviations are given in brackets (w/o = without; cat. = catalyst).

Parameter and Formulation	0	Cycle 1	Cycle 5	Cycle 10	Loss [%] 0/Cycle 1 to Cycle 10
ASE [%] (MA_27/PEG400_40) w/o cat.		74.09 (1.31)	39.05 (3.19)	34.51 (3.39)	−53.4
ASE [%] (MA_27/PEG400_40/SHP_3)		71.10 (3.55)	36.02 (3.39)	23.80 (5.14)	−66.5
ASE [%] (MA_13.4/PEG400_20) w/o cat.		68.67 (6.29)	33.24 (6.21)	28.56 (5.18)	−58.4
Bulking [%] (MA_27/PEG400_40) w/o cat.	21.84 (1.14)	16.62 (0.75)	12.44 (0.72)	11.55 (0.72)	−47.1
Bulking [%] (MA_27/PEG400_40/SHP_3)	18.37 (0.89)	13.90 (0.77)	10.31 (0.83)	7.93 (0.83)	−56.8
Bulking [%] (MA_13.4/PEG400_20) w/o cat.	14.13 (0.83)	8.07 (1.00)	5.35 (0.82)	4.34 (0.60)	−69.3
WPG [%] (MA_27/PEG400_40) w/o cat.	59.63 (5.11)	32.95 (1.06)	23.64 (0.72)	20.15 (0.63)	−66.2
WPG [%] (MA_27/PEG400_40/SHP_3)	60.64 (3.52)	33.60 (2.08)	25.88 (2.02)	20.66 (2.07)	−65.9
WPG [%] (MA_13.4/PEG400_20) w/o cat.	33.10 (4.39)	18.62 (2.55)	13.62 (2.23)	10.98 (1.92)	−66.8

**Table 9:** Influence of the curing conditions on ASE, bulking and WPG values in leaching experiments with beech wood ASE samples impregnated with water solutions of MA (13.4% [*w*/*v*]):PEG400 (20% [*w*/*v*]) (molar ratio of 2:1) without a catalyst. Standard deviations are given in brackets (w/o = without; cat. = catalyst).

Parameter and Formulation	0	Cycle 1	Cycle 5	Cycle 10	Loss [%] 0/Cycle 1 to Cycle 10
ASE [%] (MA_13.4/PEG400_20) w/o cat. 5 h/120 °C		68.67 (6.29)	33.24 (6.21)	28.56 (5.18)	−58.4
ASE [%] (MA_13.4/PEG400_20) w/o cat. 5 h/140 °C		64.41 (1.17)	32.94 (2.71)	29.43 (3.61)	−54.3
ASE [%] (MA_13.4/PEG400_20) w/o cat. 5 h/160 °C		62.38 (1.47)	39.69 (3.40)	28.48 (3.55)	−54.3
ASE [%] (MA_13.4/PEG400_20) w/o cat. 5 h/180 °C		60.52 (1.59)	38.98 (3.11)	30.26 (3.74)	−50.0
Bulking [%] (MA_13.4/PEG400_20) w/o cat. 5 h/120 °C	14.13 (0.83)	8.07 (1.00)	5.35 (0.82)	4.34 (0.60)	−69.3
Bulking [%] (MA_13.4/PEG400_20) w/o cat. 5 h/140 °C	15.03 (0.74)	10.43 (0.78)	7.54 (0.64)	8.13 (0.98)	−45.9
Bulking [%] (MA_13.4/PEG400_20) w/o cat. 5 h/160 °C	14.58 (1.08)	12.19 (0.92)	9.91 (0.70)	9.63 (0.67)	−34.0
Bulking [%] (MA_13.4/PEG400_20) w/o cat. 5 h/180 °C	10.73 (0.88)	7.99 (0.80)	5.30 (0.62)	4.45 (0.95)	−58.5
WPG [%] (MA_13.4/PEG400_20) w/o cat. 5 h/120 °C	33.10 (4.39)	18.62 (2.55)	13.62 (2.23)	10.98 (1.92)	−66.8
WPG [%] (MA_13.4/PEG400_20) w/o cat. 5 h/140 °C	27.15 (0.87)	17.45 (0.60)	12.90 (0.42)	10.91 (0.61)	−59.8
WPG [%] (MA_13.4/PEG400_20) w/o cat. 5 h/160 °C	25.56 (1.22)	19.87 (0.81)	15.87 (0.65)	13.52 (0.71)	−47.1
WPG [%] (MA_13.4/PEG400_20) w/o cat. 5 h/180 °C	21.74 (1.94)	18.44 (1.72)	14.18 (1.47)	11.51 (1.51)	−47.1

## Data Availability

The original contributions presented in this study are included in the article. Further inquiries can be directed to the corresponding author.

## Abbreviations

The following abbreviations are used in this manuscript:

PEG	Polyethylene glycol
BTCA	1,2,3,4-butanetetracarboxylic acid
CA	Citric acid
MA	Malic acid
WPG	Weight percentage gain
ASE	Anti-swelling efficiency
SHP	Sodium hypophosphite monohydrate
DMDHEU	1,3-dimethylol-4,5-dihidroxyethylene urea
cat.	Catalyst
w/o	Without
RT	Room temperature
